# A Combination of Anti-PD-L1 Treatment and Therapeutic Vaccination Facilitates Improved Retroviral Clearance via Reactivation of Highly Exhausted T Cells

**DOI:** 10.1128/mBio.02121-20

**Published:** 2021-02-02

**Authors:** Torben Knuschke, Sebastian Kollenda, Christina Wenzek, Gennadiy Zelinskyy, Philine Steinbach, Ulf Dittmer, Jan Buer, Matthias Epple, Astrid M. Westendorf

**Affiliations:** a Institute of Medical Microbiology, University Hospital Essen, University of Duisburg-Essen, Essen, Germany; b Institute of Inorganic Chemistry, Essen, Germany; c Centre for Nanointegration Duisburg-Essen (CeNIDE), Essen, Germany; d Institute for Virology, University Hospital Essen, University of Duisburg-Essen, Essen, Germany; UC Berkeley

**Keywords:** chronic retrovirus infection, T cell exhaustion, nanoparticles, T cells, checkpoint inhibition, chronic viral infection, exhaustion, immunotherapy, retroviruses, therapeutic vaccination

## Abstract

PD-1-targeted therapies have shown modest antiviral effects in preclinical models of chronic viral infection. Thus, novel therapy protocols are necessary to enhance T cell immunity and viral control to overcome T cell dysfunction and immunosuppression. Here, we demonstrate that nanoparticle-based therapeutic vaccination improved PD-1-targeted therapy during chronic infection with Friend retrovirus (FV). Prevention of inhibitory signals by blocking PD-L1 in combination with therapeutic vaccination with nanoparticles containing the microbial compound CpG and a CD8^+^ T cell Gag epitope peptide synergistically enhanced functional virus-specific CD8^+^ T cell responses and improved viral clearance. We characterized the CD8^+^ T cell populations that were affected by this combination therapy, demonstrating that new effector cells were generated and that exhausted CD8^+^ T cells were reactivated at the same time. While CD8^+^ T cells with high PD-1 (PD-1^hi^) expression turned into a large population of granzyme B-expressing CD8^+^ T cells after combination therapy, CXCR5-expressing follicular cytotoxic CD8^+^ T cells also expanded to a high degree. Thus, our study describes a very efficient approach to enhance virus control and may help us to understand the mechanisms of combination immunotherapy reactivating CD8^+^ T cell immunity. A better understanding of CD8^+^ T cell immunity during combination therapy will be important for developing efficient checkpoint therapies against chronic viral infections and cancer.

## Abstract (precis)

Despite significant efforts, vaccines are not yet available for every infectious pathogen, and the search for a protective approach to prevent the establishment of chronic infections, i.e., with HIV, continues. Immune checkpoint therapies targeting inhibitory receptors, such as PD-1, have shown impressive results against solid tumors.

## INTRODUCTION

CD8^+^ T cell dysfunction is associated with chronic viral infection (1, 2). Thus, there is a strong interest in the development of potent strategies to reinforce CD8^+^ T cell immunity to control or eradicate chronic infections. Immune checkpoint therapies (ICTs), i.e., those targeting inhibitory receptor programmed cell death-1 (PD-1) signaling, restore the cytotoxic function of CD8^+^ T cells and enhance their expansion by blocking inhibitory signals (3). However, these therapies show clinical limits that are not fully understood. Although PD-1-targeted therapies are now licensed to treat human cancers, not all patients respond equally to ICTs. Furthermore, limited efficacy in models of chronic viral infection has been reported. Thus, comprehensive investigations are under way to improve ICTs by using combined regimens.

Therapeutic vaccination aims to boost the antiviral immune response of a patient. Although previous strategies were not very successful (4–6), we have recently demonstrated that a calcium phosphate (CaP)-based nanoparticle vaccine (NPV) containing the Toll-like receptor 9 (TLR9) ligand CpG and a virus-specific CD8^+^ T cell epitope is highly efficient in activating CD8^+^ T cell immunity during chronic retroviral infection and cancer (7–9). In these studies, we demonstrated that therapeutic vaccination with this functionalized NPV facilitates a strong activation of dendritic cells (DCs) and cytotoxic CD8^+^ T cell (CTL) immunity after vaccination. Specifically, experiments in the murine Friend retrovirus (FV) model revealed that therapeutic vaccination of chronically infected mice leads to a reactivation of CTL responses and a significant drop in viral loads (7). FV is an oncogenic retroviral complex that induces lethal erythroleukemia in susceptible mouse strains. However, resistant strains, such as C57BL/6, show a robust immune response that prevents leukemia but develop a chronic infection due to virus-induced immune suppression and T cell dysfunction (10, 11). Thus, it is an excellent preclinical model for studying antiviral immunity during retroviral infection. Combinatorial immunotherapy consisting of therapeutic vaccination with CaP NPV and depletion of regulatory T cells (Tregs) during chronic FV infection leads to improved CD8^+^ T cell immunity with enhanced viral control (12). So far, it is not clear whether a combination of a PD-L1 blockade with CaP NPV might also enhance the efficacy of ICT. Therefore, we investigate whether a combination of a PD-L1 blockade with CaP nanoparticle-based therapeutic vaccination enhances viral control and characterize the CD8^+^ T cells that are affected by combination therapy during chronic retroviral infection. Recent studies have demonstrated that exhausted CD8^+^ T cells represent a pool of phenotypically and functionally heterogeneous immune cells (13, 14). For instance, specific CXCR5-expressing subsets of CD8^+^ T cells that respond to PD-1 therapy exist (15). Strikingly, the frequency and activation of these stem cell-like CXCR5^+^ follicular cytotoxic CD8^+^ T cells (T_FC_) have predictive potential for the responsiveness to PD-1 blockade (16). However, it is not known whether therapeutic vaccination is capable of expanding these highly functional cells and whether this leads to enhanced viral control in combination with PD-1 therapy. Unlike with anti-PD-1 therapy, it seems that PD-L1 antibodies are more effective in reversing the inhibitory effects of PD-1, for instance by modulating the phenotype of myeloid cells and antigen presentation in DCs (17, 18). In this study, we demonstrate that concurrent combination of a PD-L1 blockade and therapeutic vaccination has a synergistic effect on the activation of CTL immunity and viral control. Moreover, we show that this enhanced viral control underlies the generation of effector T cells as well as the rescue and reactivation of exhausted CD8^+^ T cells. T cell exhaustion itself is initiated and determined by the expression of the high mobility group (HMG) box transcription factor thymocyte selection-associated high mobility group box (TOX) (19–21). We found that high-PD-1 expressing TOX^+^ CD8^+^ T cells express granzyme B (GzmB) after combination therapy, unlike in control groups. We also describe that T_FC_ expand more strongly after combination therapy than after PD-L1 blockade alone. Importantly, we did not see synergistic effects after sequential delay of therapeutic vaccination during the PD-L1 blockade, indicating that the timing of vaccination during PD-1 therapy is highly important. Thus, our results might help us to understand the mechanisms of combination therapy during chronic viral infection and have implications for the development of novel strategies to enhance virus eradication during persistent infection.

## RESULTS

### Combination of a PD-L1 blockade and therapeutic vaccination increases virus-specific T cell immunity and virus control.

**FIG 1:**
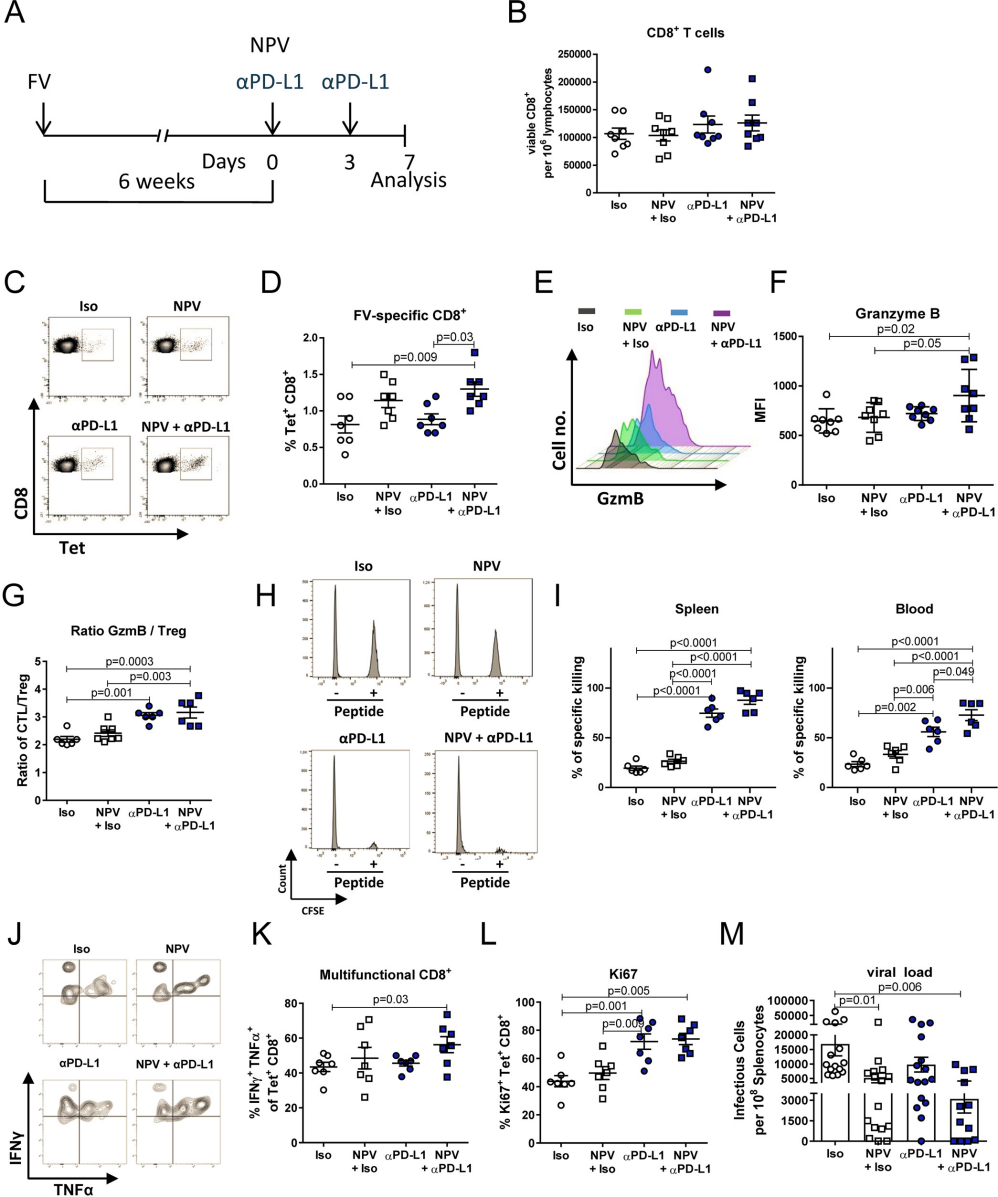
Nanoparticle-based therapeutic vaccination synergizes with a PD-L1 blockade to increase retrovirus-specific CD8^+^ T cell immunity. (A) Chronically FV-infected mice were treated twice with αPD-L1 or an isotype control (Iso) antibody, starting at 6 weeks postinfection. Groups of mice received therapeutic vaccination with CpG and GagL_85–93_-functionalized CaP nanoparticles alone or in addition to the PD-L1 blockade at the beginning of the treatment. Seven days after the initial treatment, the CD8^+^ T cell response was analyzed. (B and D) Numbers of total CD8^+^ T cells (B) or percentages of GagL_85–93_-specific tetramer^+^ CD8^+^ T cells (D) were determined in the spleen by counting viable cells using trypan blue staining. Cell counts were applied to viable-cell populations in a flow cytometric analysis. (C) Representative dot plots from flow cytometry showing the frequencies of Gag-specific tetramer^+^ CD8^+^ T cells. (E) Representative histogram from flow cytometry showing GzmB-expressing CD43^+^ tetramer^+^ CD8^+^ T cells. (F) Mean fluorescent intensity (MFI) for GzmB gated on CD43^+^ tetramer^+^ CD8^+^ T cells. (G) Ratio between GzmB-expressing CD43^+^ tetramer^+^ CD8^+^ T cells and Foxp3-expressing CD4^+^ regulatory T cells in the spleen. (H) Seven days after initial treatment, an *in vivo* cytotoxicity assay was performed to determine the killing capacity of Gag-specific CD8^+^ T cells. Representative histograms of CD45.1 gated donor cells from the spleen showing *in vivo* killing of target cells loaded with FV GagL peptide. (I) Elimination of donor CD45.1^+^ cells in the spleen and blood of chronically infected mice after treatment. (J) Representative dot plots from flow cytometry showing the frequencies of IFN-γ- and TNF-α-producing tetramer^+^ CD8^+^ T cells. (K) Frequencies of IFN-γ- and TNF-α-expressing tetramer^+^ CD8^+^ T cells after treatment. Splenocytes were restimulated *in vitro* for 4 h with PMA and ionomycin in the presence of BFA. (L) Frequencies of proliferating tetramer^+^ CD8^+^ T cells indicated by Ki67 expression. Results are pooled from two independent experiments. (M) Viral load was determined in the spleen 7 days after treatment started. Results are pooled from three independent experiments. Data shown are means ± standard errors of the means (SEM). Statistics were done by one-way ANOVA with Tukey’s multiple-comparison posttest.

To investigate whether our therapeutic vaccination strategy facilitates the effects of ICT, chronically FV-infected mice were treated with PD-L1-blocking antibodies and received concurrent vaccination with a CaP-based NPV, functionalized with TLR9 ligand CpG and a major histocompatibility complex class I (MHC I)-restricted CD8^+^ T cell Gag epitope of FV (Fig. 1A). Seven days after the NP vaccination, the overall CD8^+^ T cell response was analyzed. Although neither the PD-L1 blockade, therapeutic vaccination, nor the combination of both enhanced the number of total CD8^+^ T cells in the spleen (Fig. 1B), the combination of a PD-L1 blockade and therapeutic vaccination significantly elevated the frequencies of Gag-specific tetramer^+^ CD8^+^ T cells compared to those of untreated mice or mice given a single NPV or anti-PD-L1 (αPD-L1) treatment (Fig. 1C and D). These virus-specific CD8^+^ T cells showed increased expression of the cytotoxic molecule granzyme B (GzmB) after combination therapy compared to that after isotype control antibody or NPV treatment alone (Fig. 1E and F). At the same time, we detected increased ratios of FV-specific cytotoxic CD8^+^ T cells (CTLs) to regulatory T cells (Tregs) when mice were treated with αPD-L1 alone or in combination with NPV compared to ratios in unvaccinated mice (Fig. 1G). To directly test for improved cytotoxic activity induced by combination therapy, we performed an *in vivo* cytotoxicity assay using splenocytes loaded with the same Gag epitope of FV that we used for therapeutic vaccination. As expected, the lowest killing rate of peptide-loaded targets in the spleen was found in isotype antibody-treated control mice (∼19%), followed by an increased killing capacity after therapeutic NPV vaccination (∼26%). However, mice given αPD-L1 (∼74%) or αPD-L1 in combination with NPV (∼88%) treatment showed a significantly increased killing capacity compared to NPV- and isotype antibody-treated mice (Fig. 1H and I). Differences in the levels of killing of peptide-loaded target cells were even more obvious in the blood, as significantly more targets were killed after combination therapy than after PD-L1 blockade, NPV, or isotype antibody treatment alone (Fig. 1I). Further, we measured the ability of CD8^+^ T cells to produce the cytokines interferon gamma (IFN-γ) and tumor necrosis factor alpha (TNF-α) after *in vitro* restimulation. Importantly, FV-specific CD8^+^ T cells experienced significant functional improvement, indicated by the expression of both effector cytokines in a large proportion of cells only after combination therapy (Fig. 1J and K). Increased expression of the proliferation marker Ki67 after therapy revealed the successful activation and expansion of FV-specific CTLs, which was obviously driven by the PD-L1 blockade (Fig. 1L). We next addressed the question of whether the augmented T cell response after combination therapy resulted in superior control of chronic FV infection. We previously described that NPV alone reduces chronic FV set points but could not induce viral clearance (7). Strikingly, after combination therapy, infectious virus was undetectable in ∼30% of the mice (4 out of 13), whereas NPV treatment (12.5%) and αPD-L1 (6.25%) treatment alone led to lower virus clearance rates (Fig. 1M). Thus, combined therapy resulted in a synergistic effect on viral control in the spleen.

**FIG 2:**
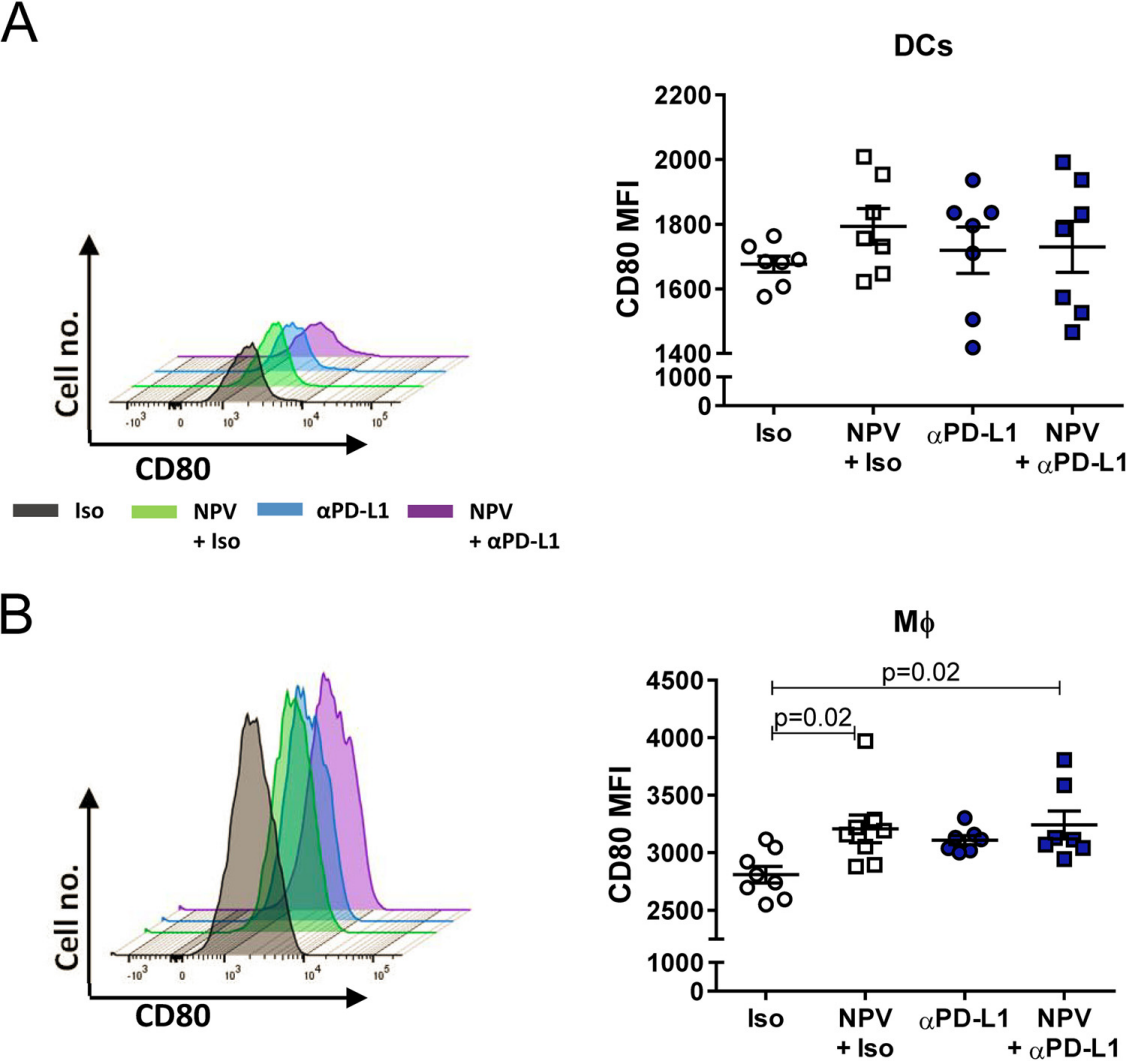
Professional APCs experience maturation by combination therapy. Chronically FV-infected mice were treated twice with αPD-L1 or an isotype control (Iso) antibody with or without therapeutic NPV. Splenocytes were analyzed 7 days after the initial treatment. Mean fluorescent intensity (MFI) data are shown for the expression of CD80 by CD11c^+^ F4/80^–^ DCs (A) and F4/80^+^ macrophages (B). The results of 2 independent experiments are illustrated. Data represent means ± SEM. Statistics were done by one-way ANOVA with Tukey’s multiple-comparisons posttest.

Antigen-specific activation of CTLs is mediated by professional antigen-presenting cells (APCs), such as dendritic cells (DCs) and macrophages. A previous study revealed the requirement of CD28 costimulation for the proliferation of exhausted T cells after PD-1-targeted therapy (22). We have recently described that CpG-functionalized CaP NPVs efficiently activate APCs, indicated by increased expression of the costimulatory B7 molecules CD80 and CD86 after *in vitro* and *in vivo* stimulation (8, 23, 24). Interestingly, activation of APCs was still enhanced in the spleen 7 days after immunization, possibly due to endocrine or paracrine effects. While CD80 was modestly upregulated by DCs only after therapeutic vaccination (Fig. 2A), we found a significantly enhanced expression by splenic macrophages after therapeutic vaccination or in combination with a PD-L1 blockade (Fig. 2B). However, no additive effect was detected compared to the effects of single treatments.

Taken together, our results indicate that the therapeutic NPV enhances the functional improvement of CD8^+^ T cells during PD-L1 blockade therapy, supported by maturation of APCs by combination therapy, which results in superior virus control.

### Combination therapy enhances the priming of effector T cells.

**FIG 3:**
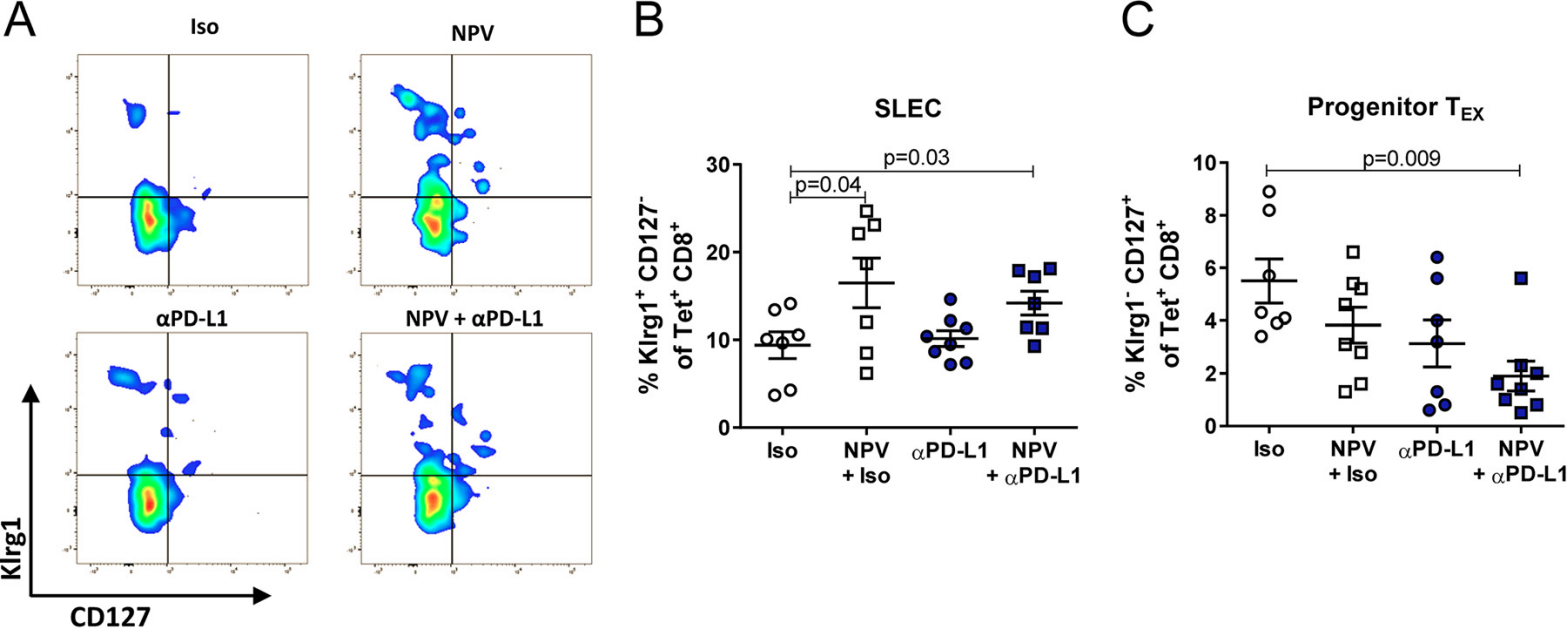
Differentiated effector cells are induced by therapeutic vaccination during chronic retrovirus infection, but not by a PD-L1 blockade. Chronically FV-infected mice were treated twice with αPD-L1 or an isotype control (Iso) antibody with or without therapeutic NPV. CD8^+^ T cell immunity was analyzed 7 days after the initial treatment. (A) Representative flow cytometry dot plots for the identification of SLECs by Klrg1 and CD127 expression. (B) Frequencies of Klrg1^+^ CD127^−^ tetramer^+^ CD8^+^ SLECs are depicted. (C) Frequencies of Klrg1^–^ CD127^+^ tetramer^+^ CD8^+^ cells are shown. The results of 2 independent experiments are illustrated. Data represent means ± SEM. Statistics were done by one-way ANOVA with Tukey’s multiple-comparison posttest. T_EX_, exhausted CD8^+^ T cells.

Since CaP NPV contained an MHC I-restricted FV epitope of the Gag protein, we wondered whether combination therapy may enhance the priming of new Gag-specific effector cells. Therefore, we analyzed the expression of the effector cell marker Klrg1 by Gag-specific Tet^+^ CD8^+^ T cells, which is induced in highly cytotoxic cells that receive strong T cell receptor signals (25). Importantly, only therapeutic vaccination alone or in combination with a PD-L1 blockade induced higher frequencies of Klrg1^+^ CD127^−^ short-lived effector cells (SLECs) among FV-specific CD8^+^ T cells than αPD-L1 treatment or controls. This result suggested that priming of new effector cells most likely occurs (Fig. 3A and B). Since we did not observe a synergistic effect compared to the effects of single treatments, it seems that only the therapeutic vaccination is responsible for the induction of new virus-specific cells. Notably, it was reported that this highly functional Klrg1^+^ SLEC population is unable to differentiate into exhausted T cells (26). However, at the same time, Klrg1^–^ CD127^+^ effector cells, which were recently described to be able to transform into exhausted T cells under chronic inflammatory conditions (2, 27), appeared significantly less frequent after combination therapy (Fig. 3C).

Thus, these results indicate that FV-specific effector CD8^+^ T cells may be generated by therapeutic vaccination during chronic retroviral infection.

### A population of highly PD-1-expressing exhausted CD8^+^ T cells experiences reactivation through combination therapy.

**FIG 4:**
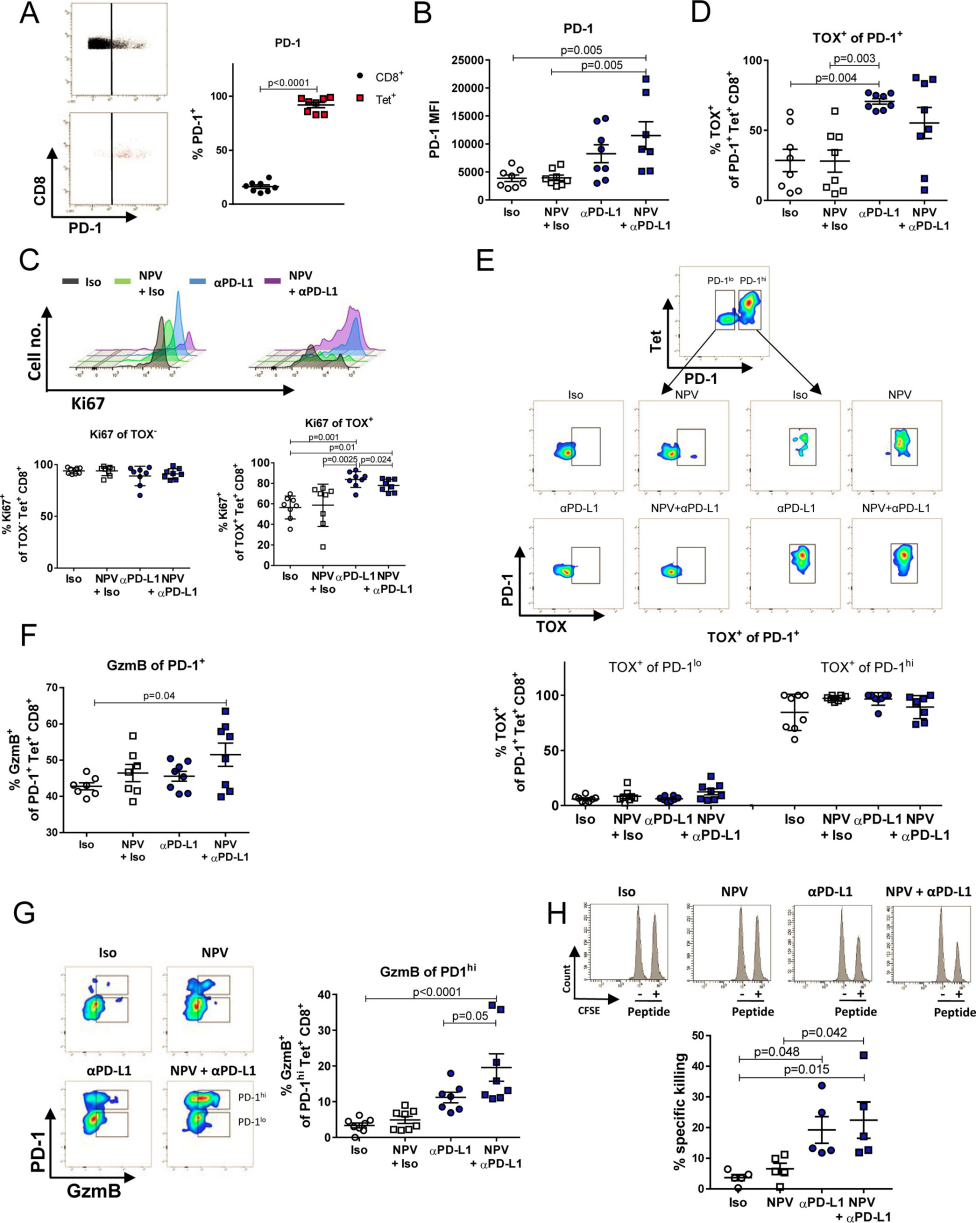
Enhanced reactivation of differentiated exhausted CD8^+^ T cells. Chronically FV-infected mice were treated twice with αPD-L1 or an isotype control (Iso) antibody with or without therapeutic NPV. CD8^+^ T cell immunity was analyzed 7 days after the initial treatment in the spleen. (A) Percentages of PD-1 expression by CD8^+^ T cells during chronic FV infection. (B) PD-1 expression by tetramer^+^ CD8^+^ T cells after treatment. (C) Percentages of Ki67-expressing TOX^+^ or TOX^–^ Tet^+^ CD8^+^ T cells. (D) Frequencies of TOX-expressing cells among PD-1^+^ tetramer^+^ CD8^+^ T cells. (E) Representative flow cytometry dot plots illustrate the expression of TOX by the two PD-1^+^ subpopulations of Tet^+^ CD8^+^ T cells. Percentages of TOX expression by PD-1^lo^ and PD-1^hi^ tetramer^+^ CD8^+^ T cells were analyzed. (F) Frequencies of GzmB-expressing PD-1^+^ tetramer^+^ CD8^+^ T cells were analyzed. (G) GzmB-expressing PD-1^hi^ tetramer^+^ CD8^+^ T cells were analyzed. Representative flow cytometry dot plots demonstrate the gating on PD-1^hi^-expressing Tet^+^ CD8^+^ T cells. (H) PD-1^hi^-expressing CD8^+^ T cells were isolated from the spleens of chronically infected mice 7 days after the initial treatment and cultured *ex vivo* with Gag peptide-loaded and unloaded CFSE-stained target cells. The specific killing of peptide-loaded target cells is depicted. The results from 2 independent experiments were pooled. Bars represent means ± SEM. Statistical analysis was performed by one-way ANOVA with Tukey’s multiple-comparison posttest.

T cell dysfunction is associated with high expression of PD-1 during chronic infections. Notably, PD-1-expressing cells are phenotypically and functionally heterogeneous immune cells. For instance, it has been reported that PD-1^hi^ CD8^+^ T cells are not able to restore functional properties by PD-1-targeted therapy during chronic viral infection (13). However, reactivation of these cells seems to be possible since the depletion of Tregs confers enhanced functionality (28). The majority of Gag-specific CD8^+^ T cells expressed PD-1 during chronic FV infection (Fig. 4A), and PD-1 expression further increased after the PD-L1 blockade (Fig. 4B). Surprisingly, Gag-specific CD8^+^ T cells expressed the highest level of PD-1 after combination therapy. To determine whether these cells were also highly exhausted, we investigated the expression of the transcription factor TOX by PD-1^+^ virus-specific CD8^+^ T cells. TOX has been identified in murine lymphocytic choriomeningitis virus (LCMV) infection as a central regulator of T cell exhaustion, as it represses terminal effector T cell-specific epigenetic events while initiating key epigenetic changes for exhausted T cells (19, 20). These TOX-expressing Gag-specific CD8^+^ T cells still have a proliferative potential, as they responded to a PD-L1 blockade with increased Ki67 expression compared to that after treatment with isotype control antibody or NPV (Fig. 4C). Notably, while we found TOX expression to be significantly upregulated by PD-1^+^ Gag-specific CD8^+^ T cells after a PD-L1 blockade, there was no such enhanced expression after therapeutic vaccination alone or combination therapy (Fig. 4D). However, high frequencies of PD-1^hi^ cells expressed TOX, while the PD-1^lo^ population showed lower TOX expression levels (Fig. 4E), supporting the idea that PD-1^hi^ cells are more terminally differentiated than exhausted T cells. However, despite the increased PD-1 expression, virus-specific CD8^+^ T cells were highly capable of expressing GzmB after combination therapy (Fig. 4F). In more detail, we identified that especially the PD-1^hi^ population responded after combination therapy with GzmB expression (Fig. 4G). In order for us to proof their cytotoxic capacity, an *ex vivo* CTL killing assay was performed. PD-1^hi^ CD8^+^ T cells were isolated from mice and cultured with splenocytes loaded with the Gag epitope of FV. The killing of peptide-loaded splenocytes by CD8^+^ PD-1^hi^ cells from mice receiving combination therapy was significantly higher than with NPV alone or isotype antibody treatment (Fig. 4H).

Taken together, the results indicate that although the frequencies of PD-1-expressing CD8^+^ T cells proceeded after the PD-L1 blockade and combination therapy, therapeutic vaccination induces robust responses of the apparently exhausted CTLs, although they express high levels of PD-1.

### CXCR5^+^ follicular cytotoxic CD8^+^ T cells expand and are highly activated after the combination of a PD-L1 blockade and therapeutic vaccination.

**FIG 5:**
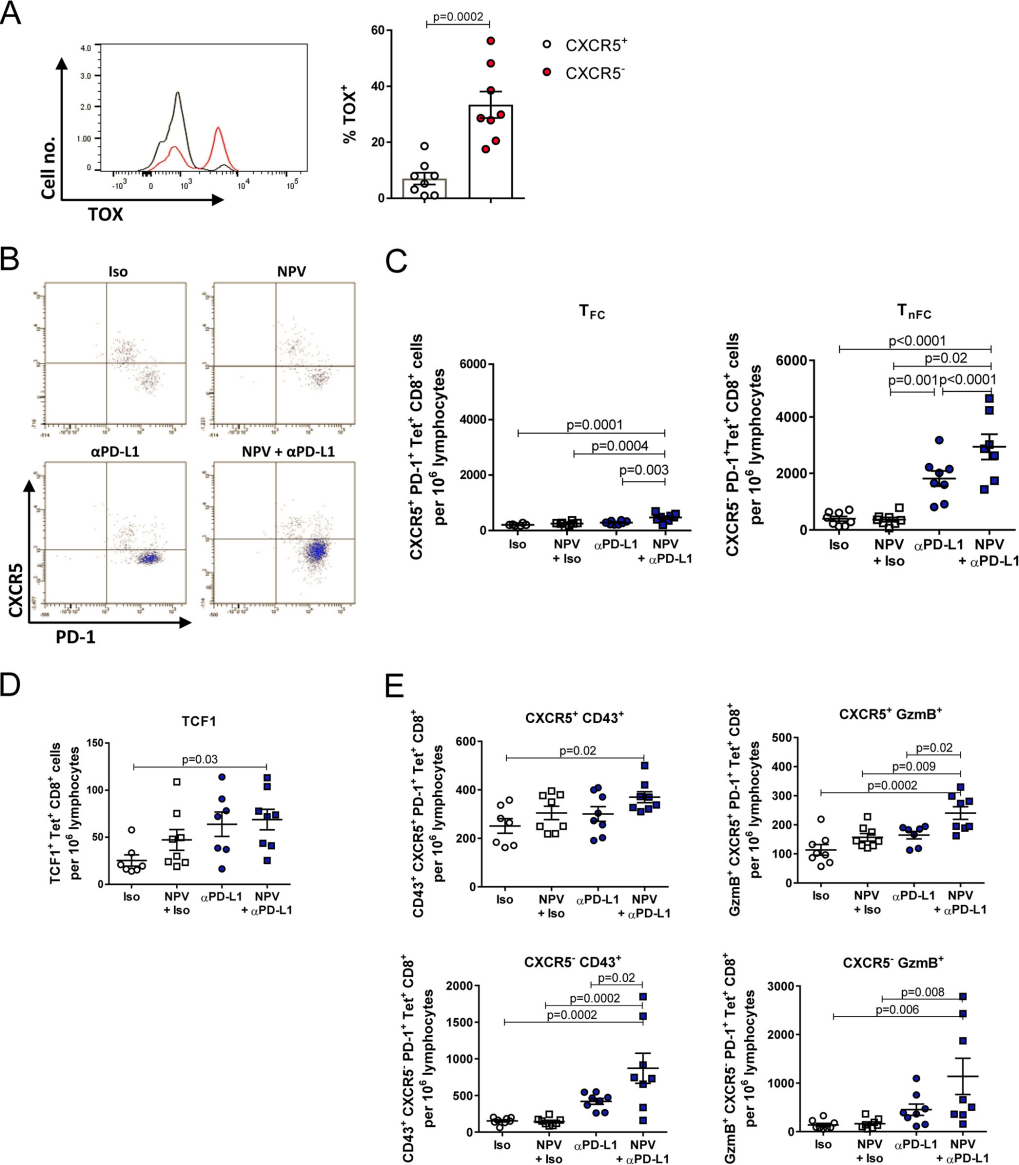
Combination therapy synergistically enhances the reactivation of CXCR5^+^ follicular cytotoxic CD8^+^ T cells. Chronically FV-infected mice were treated as described in the text. CD8^+^ T cell immunity was analyzed 7 days after the initial treatment. (A) Frequencies of TOX-expressing PD-1^+^ CXCR5^+^ or CXCR5^–^ tetramer^+^ CD8^+^ T cells. (B) Representative dot plots showing CXCR5^+^ PD-1^+^ tetramer^+^ CD8^+^ T cell populations during chronic FV infection. (C) Numbers of follicular CXCR5^+^ and nonfollicular CXCR5^–^ cytotoxic tetramer^+^ CD8^+^ T cells are depicted. (D) Numbers of TCF1^+^ tetramer^+^ CD8^+^ T cells. (E) Numbers of CD43- or GzmB-expressing CXCR5^+^ and CXCR5^–^ tetramer^+^ CD8^+^ T cells are shown. The results of 2 independent experiments are illustrated. Data represent means ± SEM. Statistical analysis was performed by one-way ANOVA with Tukey’s multiple-comparison posttest.

Recent studies have reported the emergence of CD8^+^ T cells expressing the chemokine receptor CXCR5 during chronic viral infection (29). In fact, these PD-1-expressing cells were identified to strongly respond during PD-1-targeted therapy, in contrast to the more differentiated PD-1^hi^ exhausted T cells (15, 30). In addition, we found that TOX was expressed mainly by CXCR5^–^ CD8^+^ T cells, whereas the stem cell-like CXCR5^+^ CD8^+^ T cells did indicate that they are less exhausted (Fig. 5A). To evaluate whether therapeutic vaccination could further enhance the expansion and activation of these T_FC_ in addition to the PD-L1 blockade, we determined the magnitude of Gag-specific tetramer-positive (tetramer^+^) CD8^+^ T cells and their coexpression of PD-1 and CXCR5. Interestingly, numbers of T_FC_ synergistically expanded after combination therapy compared to numbers after therapeutic vaccination or PD-L1 blockade alone (Fig. 5B and C). In line with this, frequencies of TCF1-expressing Gag-specific CD8^+^ T cells increased after combination therapy compared to frequencies in untreated mice (Fig. 5D). TCF1 was described to maintain stemness and sustain CD8^+^ T cell responses during chronic LCMV infection (15, 26). As expected, the CXCR5^–^ nonfollicular cytotoxic CD8^+^ T cells (T_nFC_) likewise expanded after blockade of PD-L1, as these cells were described to arise from CXCR5^+^ CD8^+^ T cells with enhanced functionality. However, the combinatorial treatment synergistically increased the number of T_nFC_ (Fig. 5C). Moreover, we found more fully activated CD43- and GzmB-expressing T_FC_ and T_nFC_ after combination therapy than after NPV or αPD-L1 treatment alone (Fig. 5D). Overall, relative numbers of T_nFC_ were markedly higher than T_FC_ numbers.

Together, these results demonstrate that therapeutic vaccination with CaP NP highly increases the numbers of functional T_FC_ and T_nFC_ during concurrent αPD-L1 treatment of chronic retroviral infection.

### The timing of therapeutic vaccination during a PD-L1 blockade is critical for effective CD8^+^ T cell reactivation.

**FIG 6:**
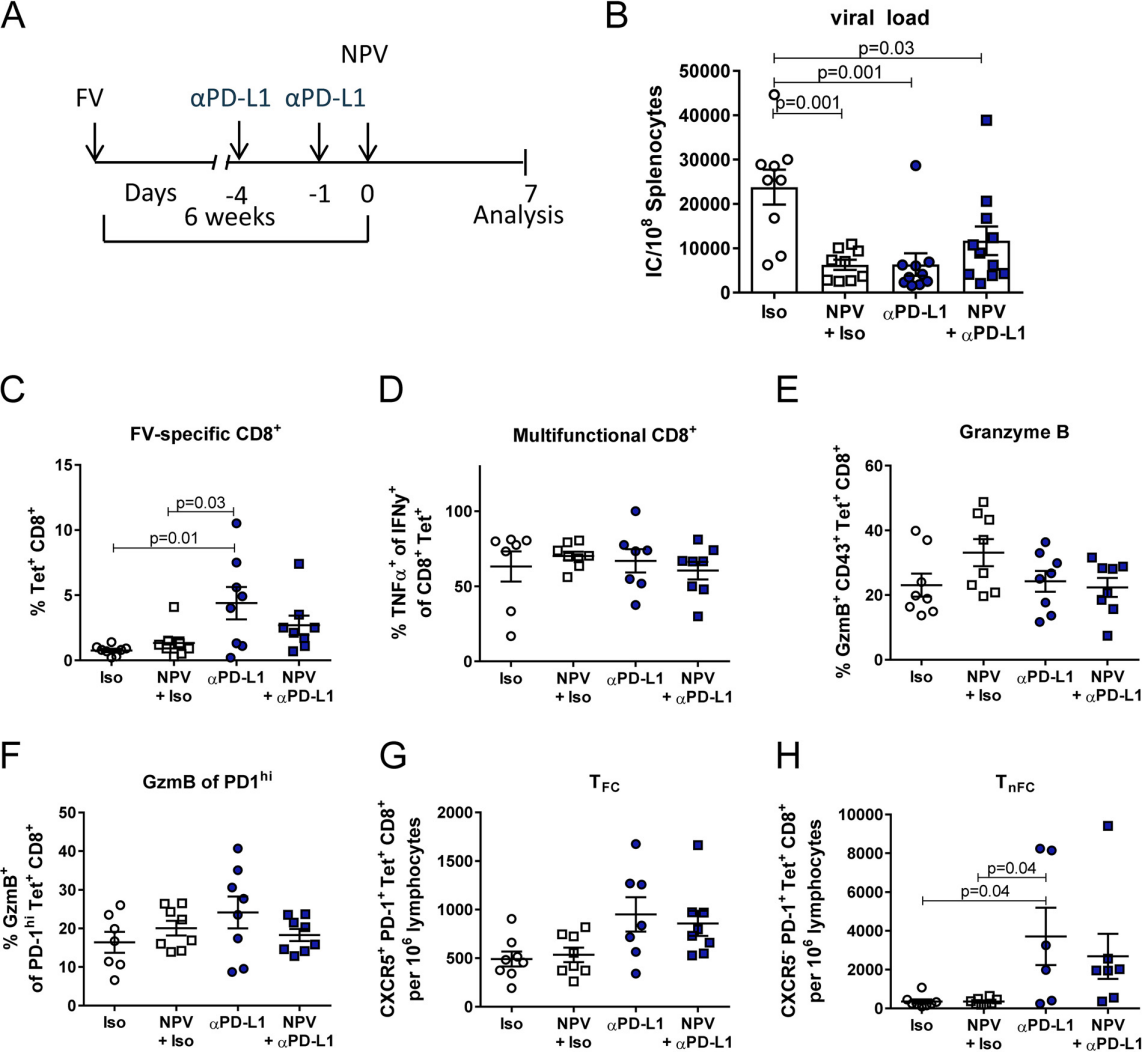
The timing of therapeutic vaccination is critical for effective combination therapy with a PD-L1 blockade. (A) Chronically FV-infected mice were treated twice with αPD-L1 or an isotype control (Iso) antibody starting at 6 weeks postinfection. Groups of mice received either therapeutic vaccination with CpG- and GagL_85–93_-functionalized CaP nanoparticles alone or in addition to a PD-L1 blockade 4 days after the treatment start. Seven days after therapeutic vaccination, the CD8^+^ T cell response was analyzed. (B) Viral load was determined in the spleen 7 days after therapeutic vaccination. (C) Percentages of Gag-specific tetramer^+^ CD8^+^ T cells are shown. (D) Frequencies of IFN-γ- and TNF-α-expressing tetramer^+^ CD8^+^ T cells after treatment. Splenocytes were restimulated *in vitro* for 4 h. (E) Frequencies of GzmB-expressing CD43^+^ tetramer^+^ CD8^+^ T cells. (F) Frequencies of GzmB-expressing PD-1^hi^ tetramer^+^ CD8^+^ T cells. Numbers of CXCR5^+^ (G) or CXCR5^–^ (H) tetramer^+^ CD8^+^ T cells are shown. The results of 2 independent experiments were pooled. Bars represent means ± SEM. Statistical analysis was performed by one-way ANOVA with Tukey’s multiple-comparison posttest.

Given the augmented proliferation of FV-specific CD8^+^ T cells by a PD-L1 blockade, we evaluated whether additional therapeutic vaccination might be more effective if it was given after the expansion of CTLs induced by αPD-L1 treatment. In this regard, mice were therapeutically vaccinated with CaP NP 4 days after the initial PD-L1 blockade (Fig. 6A). Interestingly, when therapeutic vaccination was delayed in combination with a PD-L1 blockade, we did not detect a synergistic effect on viral control compared to the effects of concurrent combination therapy (Fig. 6B). To understand why the sequential delay of therapeutic vaccination during PD-L1 blocking therapy led to a diminished viral clearance, we analyzed the CD8^+^ T cells in more detail. Importantly, delayed therapeutic vaccination in combination with a PD-L1 blockade did not enhance frequencies of Gag-specific tetramer^+^ CD8^+^ T cells compared to frequencies with therapeutic vaccination or a PD-L1 blockade, nor did it lead to functional improvement, indicated by unchanged IFN-γ and TNF-α expression (Fig. 6C and D). Moreover, delayed therapeutic vaccination during a PD-L1 blockade did not enhance the frequencies of GzmB-expressing CD8^+^ T cells (Fig. 6E). Importantly, GzmB expression was also not enhanced by differentiated PD-1^hi^-expressing CD8^+^ T cells after concurrent combination therapy (Fig. 6F). There was also no synergistic effect on the expansion of T_FC_ and T_nFC_ compared to that after PD-L1 blockade alone (Fig. 6G and F).

In summary, the results demonstrate that, unlike with concurrent combination therapy, delayed therapeutic vaccination during PD-L1 blockade does not lead to synergistic enhancement of CD8^+^ T cell immunity and therefore has no synergistic effect on viral control.

## DISCUSSION

Novel strategies for therapeutic treatment of chronic viral infections are still of high relevance, as a protective vaccine against, e.g., HIV is not available yet. Persistent viral infection during hepatitis B virus (HBV) or HIV infection results in T cell dysfunction. One of the central regulators of T cell dysfunction is PD-1. PD-1-targeted therapies exhibited tremendous impact on T cell immunity during chronic viral infection and several types of cancer (31, 32). However, some patients do not respond to PD-1-targeted therapy. Especially, the responsiveness during chronic viral infection is limited (33–36), indicating that new regimens are necessary to exploit the full potential of anti-PD-1 therapy. In this study, we evaluated the efficacy of a PD-L1 blockade in combination with CpG-containing CaP nanoparticle-based therapeutic vaccination and characterized the affected CD8^+^ T cell subsets. PD-L1 blockade in combination with concurrent but not sequential delayed therapeutic vaccination enhanced the expansion of virus-specific CD8^+^ T cells during chronic retrovirus infection. In addition, concurrent therapeutic vaccination improved even functional capacities of differentiated PD-1^hi^ exhausted CD8^+^ T cells. The combination therapy synergistically enhanced viral control compared to that after therapeutic vaccination or a PD-L1 blockade alone. Thus, we describe the impact of combination immunotherapy on the reactivation of exhausted CD8^+^ T cells. Our strategy may be useful for the development of effective regimens against chronic viral infection and cancer.

PD-1 is significantly upregulated on virus-specific CD8^+^ T cells during chronic viral infection, e.g., HIV (37, 38). Of note, the proportion of CD8^+^ T cells expressing PD-1 strongly correlates with plasma viral load levels (38). Therefore, PD-1 expression seems to be an indicator of disease progression during persistent infection. Indeed, we found that PD-1 expression is upregulated on CD8^+^ T cells during chronic FV infection. Especially among the virus-specific CD8^+^ T cell population, there are high frequencies of PD-1-expressing cells. These findings are applicable to other disease models, such as cancer, in which CD8^+^ T cells are as well continuously exposed to antigenic stimulation (39). Thus, PD-1-targeted therapies blocking either PD-1 or PD-L1 have revolutionized cancer immunotherapy. However, these therapies have clinical limitations that are not well understood yet. Importantly, additional blockade of other inhibitory receptors in combination with αPD-L1 strongly increases the reactivation of T cell immunity during FV infection, promoting virus control, but may even enhance the risk of fatal systemic immunopathology (40, 41). Hence, other strategies should be considered to increase the responsiveness to PD-1 therapy. One hint is given by the composition of the gut microbiota influencing the host response to PD-1 therapy (42). Combining a blockade of PD-L1 with therapies containing specific microbial products might therefore improve a therapeutic effect. A recent study already described that LPS-induced TLR4 signaling enhances the effect of PD-1 blockade therapy during chronic LCMV infection (43). Recently, we have demonstrated that therapeutic vaccination with a nanoparticle-based vaccine containing bacterially derived CpG motifs and a CD8^+^ T cell-restricted Gag epitope during chronic FV infection leads to enhanced activation of the CD8^+^ T cell response, which is driven by CpG-induced type I interferons (IFN I) (8). However, whether this strategy might boost the response to αPD-L1 therapy remained unknown. As shown in this study, combination therapy had an additive effect on the function of epitope-specific CD8^+^ T cells, indicated by enhanced GzmB and cytokine expression, as well as on viral control in the spleen compared to the effects of NPV or αPD-L1 treatment alone. This was consistent with the ability to significantly kill more FV-antigen loaded target cells *in vivo* after combination therapy than after any single treatment. We found that the therapeutic vaccination strategy induced terminally differentiated Klrg1^+^ effector cells with or without PD-L1 blockade. As these cells develop during the process of naive T cell priming, these effector cells might enhance the antiviral effect of the combinatorial treatment compared to the effect of the PD-L1 blockade alone. Also, others have described the induction of SLECs during therapeutic vaccination, which can be critical for viral clearance (44, 45). As we detected synergistic effects through combination therapy on CTL reactivation, NPV-induced IFN I might also enhance the effects of combinatorial treatment. However, a previous study could already exclude the necessity of IFN I signaling for the synergistic effect during LPS-containing anti-PD-1 therapy (43). Multiple epitopes or other microbial products for vaccination regimens may be more effective in reducing viral load by combining a PD-L1 blockade with therapeutic vaccination. Consistently with the enhanced killing capacity, we also recognized an enhanced ratio between GzmB-expressing CD8^+^ T cells and Tregs upon combination therapy. Increased Treg frequencies are a hallmark of immune dysfunction during chronic viral infection. Depletion of Tregs during chronic FV infection with or without therapeutic vaccination increases the frequency and function of CTLs, demonstrating that T cell dysfunction is also associated with Treg responses (12, 28). Combining therapeutic vaccination and a blockade of PD-1 signaling together with manipulation of the Treg population might therefore further enhance the observed CTL reactivation. Importantly, we found that our approach, besides generating terminally differentiated effector cells, strongly reinforced exhausted CD8^+^ T cells. Recent studies have demonstrated that exhausted T cells are phenotypically and functionally heterogeneous immune cells (13, 14, 46). Interestingly, while it has been shown that PD-1^hi^ CD8^+^ T cells do not respond to PD-1-targeted therapy, we found GzmB expression to be highly upregulated in a distinct PD-1^hi^ population of FV-specific CD8^+^ T cells after combination therapy, but not after therapeutic vaccination or PD-L1 blockade alone. Importantly, we were able to demonstrate that these cells can efficiently kill FV antigen-pulsed target cells *in vitro*. Although there was no big difference detectable between PD-1^hi^-expressing CD8^+^ T cells from αPD-L1-treated mice compared to those from mice that received the combination therapy, frequencies of GzmB-expressing PD-1^hi^ Tet^+^ CD8^+^ T cells were enhanced in these mice, demonstrating that the cytotoxicities per cell might be comparable, but the enhanced expansion of CTLs after combination therapy is decisive. Since it has been demonstrated that sufficient costimulation of CD8^+^ T cells is a prerequisite for the restoration of effector cell function during a PD-1 blockade (22), the NPV-induced activation of innate immune cells, such as DCs and macrophages, that we observed may be essential for this effect. Actually, a specific subset of PD-1-expressing CD8^+^ T cells expands upon PD-L1 blockade therapy during chronic LCMV infection, which likewise supports the idea that disease progression might influence the responsiveness to PD-1 therapy. In this study, a PD-L1 blockade with or without therapeutic vaccination during chronic FV infection increased the expression of PD-1 by virus-specific CD8^+^ T cells 7 days after initial treatment. Several studies confirmed that a CXCR5-expressing population of exhausted CD8^+^ T cells with stem cell-like properties selectively proliferate and differentiate in response to PD-1 therapy (15, 29). However, very recently, it has been reported that during this process, the stem cell-like population gives rise to highly functional transitory CXCR5^–^ cells with an effector-like phenotype, which finally convert into more differentiated exhausted CD8^+^ T cells with progressed exhaustion (30). Thus, for the improvement of PD-1-targeted therapy it is of interest to examine whether only the stem cell-like CD8^+^ T cells can still respond to combination therapy or whether differentiated exhausted T cells can be reinforced. Interestingly, we observed activated CXCR5^+^ T_FC_ to be enhanced synergistically after combination therapy during chronic FV infection, which may (i) increase the responsiveness to a PD-L1 blockade and (ii) demonstrate an additional activation of these less exhausted cells by therapeutic vaccination, leading to an increased conversion to CXCR5^–^ transitory effector cells. Since therapeutic vaccination was administered concurrently with a PD-L1 blockade, this might have an impact on the emerging transitory effector cells in particular. Consistently, after combination therapy, we also detected an expansion of differentiated CXCR5^–^ PD-1^hi^ CD8^+^ T cells that were fully activated with high potential to produce GzmB. At the same time point, viral loads were decreased. It would be very interesting to identify whether T cell exhaustion appears to be less severe after an additional 7 days or if an extension of the combination therapy eventually leads to a correlation of PD-1 expression and viral load. Of note, TOX was not directly associated with PD-1 expression after combination therapy, unlike with αPD-L1 therapy, which may indicate that the progression of terminal exhaustion is delayed under combination therapy with increased responsiveness. Although TOX expression was described to be strongly associated with an exhausted phenotype of CD8^+^ T cells (19–21, 47), it was recently described that TOX is also a universal regulator of human memory CD8^+^ T cells specific for chronic viruses (48). In line with this, PD-1 signaling seems also to be important for CD8^+^ T cell memory formation (49). Thus, TOX and PD-1 might not only be involved in signaling that finally leads to T cell exhaustion limiting CD8^+^ T cell function. Indeed, we observed that TOX-expressing FV-specific CD8^+^ T cells had an increased proliferative potential after PD-1 therapy.

One major concern of combination therapies is the timing of additional treatment with PD-1 therapy. For instance, it has been shown that during chronic LCMV and HBV infection, high viral loads and severe T cell dysfunction are associated with poor responses to therapeutic vaccination (6, 36). Thus, when T cell exhaustion is less severe, therapeutic vaccination may be more efficient. However, although a PD-L1 blockade during chronic infection enhanced viral control in our model, the additional sequential delayed therapeutic vaccination did not lead to synergistic effects on the CD8^+^ T cell immunity. In more detail, delayed therapeutic vaccination resulted in no synergistic effects on the activation of CTLs in general or on the reactivation of exhausted T cells specifically. This may be due to a lower impact of the delayed vaccination on emerging transitory effector cells during the conversion of CXCR5^+^ stem cell-like exhausted CD8^+^ T cells under αPD-L1 therapy. A previous study demonstrated that the timing of a PD-1 blockade is also important during OX40-targeted cancer immunotherapy due to OX40-induced enhanced expression of other inhibitory receptors (50). Interestingly, we did not observe any upregulation of additional inhibitory receptors, such as CD103, CD160, or TIM-3, induced by αPD-L1 treatment (data not shown). Thus, our results suggest that the timing of the different components of a combination therapy may be important to efficiently hit the emerging functional transitory CD8^+^ T cells.

In summary, our study shows that a PD-L1 blockade during chronic retroviral infection in combination with a CpG-containing nanoparticle-based therapeutic vaccination strategy synergistically boosts virus-specific CD8^+^ T cell immunity and promotes viral control. We demonstrate that our strategy enhances especially the functional capacity of exhausted CD8^+^ T cells. This strategy may enhance the treatment of chronic viral infections and may also improve cancer therapy.

## MATERIALS AND METHODS

Animal experiments were performed in strict accordance with the German regulations of the Society for Laboratory Animal Science (GV-SOLAS) and the European Health Law of the Federation of Laboratory Animal Science Associations (FELASA). The protocol was approved by the North Rhine-Westphalia State Agency for Nature, Environment and Consumer Protection (LANUV). All efforts were made to minimize suffering.

### Mice.

Female C57BL/6 mice were purchased from Envigo Laboratories (Envigo CRS GmbH, Rossdorf, Germany). All mice used in the experiments were 8 to 10 weeks old at the time point of infection and housed under specific-pathogen-free conditions in the Laboratory Animal Facility of the University Hospital Essen.

### Cells and cell culture.

A murine fibroblast cell line from Mus dunni (51) was maintained in Roswell Park Memorial Institute (RPMI) medium containing 10% endotoxin-free fetal calf serum (FCS) and 50 μg ml^−1^ penicillin/streptomycin. Cell lines were maintained in a humidified 5% CO_2_ atmosphere at 37°C.

### TLR ligand and viral peptides.

The phosphorothioate-modified class B CpG 1826 was purchased from Eurofins MWG Operon. The FV protein-derived Gag and gp70 peptide sequences containing MHC I and MHC II epitopes were synthesized with the following sequences: GagL_85-93_, CCLCLTVFL, and gp70_123–141_, EPLTSLTPRCNTAWNRLKL (JPT Peptide Technologies GmbH). The cysteine in the GagL peptide sequence was replaced with aminobutyric acid.

### Antibodies and flow cytometry.

The following monoclonal antibodies were used. αCD4 (clone RM4-5), αCD8 (clone 53-6.7), αCD80 (clone 16-10A1), αPD-1 (clone J43), and αCD43 (clone 1B11) were obtained from BD Biosciences Pharmingen. αGzmB (clone GB12) and αTOX (clone TXRX10) antibodies were purchased from Invitrogen. αFoxp3 (clone FJK-16s), Ki67 (clone SolA15), and CD127 (clone A7R34) antibodies were purchased from eBioscience. αIFN-y (clone XMG1.2), αTNF-α (clone MP6-XT22), αTCF1 (clone 7F11A10), and CXCR5 (clone L138D7) antibodies were obtained from BioLegend. To detect Friend virus-specific CD8^+^ T cells, a phycoerythrin (PE)-conjugated recombinant MHC I H2-D^b^ tetramer (Beckman Coulter) specific for the FV GagL peptide was used (52). Intracellular staining for GzmB, Foxp3, IFN-γ, TNF-α, and Ki67 was performed as described previously (8). Splenocytes were restimulated for 4 h with phorbol myristate acetate (PMA) and ionomycin in the presence of brefeldin A (BFA). Intracellular staining for TOX was performed according to the Foxp3 staining protocol. For all stainings, a fixable viability dye (eBioscience) was used to exclude dead cells from analysis. Data were acquired by using an LSR II instrument using DIVA software (BD Biosciences Pharmingen).

### Friend virus and chronic infection.

A lactate dehydrogenase-elevating virus (LDV) B cell-tropic, polycythemia-inducing Friend virus complex (FV) was obtained from BALB/c mouse spleen cell homogenate 14 days postinfection (53). To induce chronic FV infection, naive FV-resistant CB57BL/6 mice were infected intravenously (i.v.) with 15,000 spleen focus-forming units (SFFU).

### *In vivo* antibody blockade and therapeutic vaccination.

PD-L1 was blocked by intraperitoneal injection of 200 μg of anti-mouse PD-L1 antibody (clone 10F:9G2) or IgG2b isotype control 2 times every 3 days, beginning 42 days after infection. For therapeutic vaccination, mice were injected once subcutaneously either with 100 μl phosphate-buffered saline (PBS) or CaP NPs functionalized with CpG/GagL/gp70 (10.3 μM CpG and 40.8 μg ml^−1^ GagL/gp70 peptide or with CaP/CpG/GagL/gp70/CaP/CpG) in both hind footpads (50 μl each) on the day of the initial PD-L1 blockade. Nanoparticles were prepared as described previously (8).

### *In vivo* and *ex vivo* cytotoxicity assay.

CTL killing assays were performed as described previously to test the cytotoxic capacity (54). The protocol was modified to measure cytotoxicity in FV-infected mice. Briefly, splenocytes from a naive CD45.1 mouse were loaded with 1 μM GagL peptide. The peptide-loaded cells were stained with 200 nM CFSE (carboxyfluorescein succinimidyl ester; Molecular Probes). As a reference, nonstained splenocytes of a CD45.1 mouse were used. Splenocytes (1 × 10^7^ cells of each population) were transferred i.v. into naive or chronically FV-infected mice 7 days after the initial start of treatment. One hour after adoptive transfer, spleen and blood from recipient mice were harvested. Donor cells were distinguished from recipient cells and from one another based on expression of CD45.1 and CFSE via flow cytometry. The percentage of killing of each population of peptide-loaded cells was calculated as follows: [1 − (percentage of peptide-loaded/percentage of unloaded chronically FV-infected cells) × (percentage of peptide-loaded/percentage of unloaded naive cells) × 100%].

For the analysis of the cytotoxicity of PD-1^hi^ CTLs, PD-1^hi^ CD8^+^ T cells were isolated by flow cytometry-based separation from the spleens of chronically FV-infected mice 7 days after the initial treatment. Splenocytes from a naive mouse were loaded with GagL peptide and stained with CFSE as described above (CFSE^hi^). Unloaded cells (CFSE^lo^) were stained with 4 nM CFSE as a reference. PD-1^hi^ CD8^+^ cells (5 × 10^4^) from treated chronically FV-infected mice or CD8^+^ T cells from naive mice were cultured with peptide-loaded and unloaded splenocytes (5 × 10^4^ of each population) for 12 h at 37°C.

### Infectious-center assay.

Determination of viral loads by infectious-center assay was performed as described previously (55). Spleens were cropped and rinsed with RPMI medium containing 10% FCS and 50 μg ml^−1^ penicillin/streptomycin. Spleen cells were counted and serially diluted before they were seeded onto Mus dunni cells, and then they were incubated under standard tissue culture conditions for 3 days, fixed with ethanol, and labeled with the primary F-MuLV Env-specific monoclonal antibody 720 (56). After the cells were washed, a secondary horseradish peroxidase (HRP)-conjugated rabbit anti-mouse Ig antibody (Dako) was added. Foci representing infectious centers were detectable after the addition of aminoethylcarbazole (Sigma-Aldrich) as the substrate for HRP. Foci were counted and numbers of infectious centers (IC) per spleen were calculated.

### Statistical analysis.

Statistical analysis was performed by using Student's *t* test or one-way analysis of variance (ANOVA) to compare multiple groups using Tukey’s multiple-comparison test. Data analysis was performed using Prism 7.0 software (GraphPad). Statistical significance was set at the level of a *P* of <0.05.
